# Poisonous Ornaments

**Published:** 1873-06

**Authors:** 


### POISONOUS ORNAMENTS.

It is safest and best that every green thing should be avoided as a painted plaything for children, as green paints or coloring matters contain arsenic or verdigris, both of which are rank poisons. A little girl was amusing herself one day with a bunch of toy green grapes. She picked one off and sucked it; the next day she was dead. Another child's toy cupboard was lined with green paper; in a few days the little thing sickened and died. On a chemical examination, a bit of the paper, six inches square, yielded twelve grains of the arsenite of copper, enough to kill two grown persons.

